# Defining Misinformation and Related Terms in Health-Related Literature: Scoping Review

**DOI:** 10.2196/45731

**Published:** 2023-08-09

**Authors:** Ibrahim K El Mikati, Reem Hoteit, Tarek Harb, Ola El Zein, Thomas Piggott, Jad Melki, Reem A Mustafa, Elie A Akl

**Affiliations:** 1 Outcomes and Implementation Research Unit Department of Internal Medicine University of Kansas Medical Center Kansas City, KS United States; 2 Clinical Research Institute, Faculty of Medicine American University of Beirut Beirut Lebanon; 3 Division of Cardiology, Department of Medicine Johns Hopkins University School of Medicine Baltimore, MD United States; 4 University Libraries American University of Beirut Beirut Lebanon; 5 Department of Health Research Methods, Evidence, and Impact McMaster University Hamilton, ON Canada; 6 Department of Family Medicine Queens University Kingston, ON Canada; 7 Institute of Media Research and Training Lebanese American University Beirut Lebanon; 8 Department of Internal Medicine American University of Beirut Beirut Lebanon

**Keywords:** misinformation, disinformation, infodemic, fake news, malinformation, health, COVID-19, scoping review, health-related literature, electronic database, misleading, related term, systematic review

## Abstract

**Background:**

Misinformation poses a serious challenge to clinical and policy decision-making in the health field. The COVID-19 pandemic amplified interest in misinformation and related terms and witnessed a proliferation of definitions.

**Objective:**

We aim to assess the definitions of misinformation and related terms used in health-related literature.

**Methods:**

We conducted a scoping review of systematic reviews by searching Ovid MEDLINE, Embase, Cochrane, and Epistemonikos databases for articles published within the last 5 years up till March 2023. Eligible studies were systematic reviews that stated misinformation or related terms as part of their objectives, conducted a systematic search of at least one database, and reported at least 1 definition for misinformation or related terms. We extracted definitions for the terms misinformation, disinformation, fake news, infodemic, and malinformation. Within each definition, we identified concepts and mapped them across misinformation-related terms.

**Results:**

We included 41 eligible systematic reviews, out of which 32 (78%) reviews addressed the topic of public health emergencies (including the COVID-19 pandemic) and contained 75 definitions for misinformation and related terms. The definitions consisted of 20 for misinformation, 19 for disinformation, 10 for fake news, 24 for infodemic, and 2 for malinformation. “False/inaccurate/incorrect” was mentioned in 15 of 20 definitions of misinformation, 13 of 19 definitions of disinformation, 5 of 10 definitions of fake news, 6 of 24 definitions of infodemic, and 0 of 2 definitions of malinformation. Infodemic had 19 of 24 definitions addressing “information overload” and malinformation had 2 of 2 definitions with “accurate” and 1 definition “used in the wrong context.” Out of all the definitions, 56 (75%) were referenced from other sources.

**Conclusions:**

While the definitions of misinformation and related terms in the health field had inconstancies and variability, they were largely consistent. Inconstancies related to the intentionality in misinformation definitions (7 definitions mention “unintentional,” while 5 definitions have “intentional”). They also related to the content of infodemic (9 definitions mention “valid and invalid info,” while 6 definitions have “false/inaccurate/incorrect”). The inclusion of concepts such as “intentional” may be difficult to operationalize as it is difficult to ascertain one’s intentions. This scoping review has the strength of using a systematic method for retrieving articles but does not cover all definitions in the extant literature outside the field of health. This scoping review of the health literature identified several definitions for misinformation and related terms, which showed variability and included concepts that are difficult to operationalize. Health practitioners need to exert caution before labeling a piece of information as misinformation or any other related term and only do so after ascertaining accurateness and sometimes intentionality. Additional efforts are needed to allow future consensus around clear and operational definitions.

## Introduction

Misinformation has long plagued both the public sphere and the scientific community, but it has become particularly ubiquitous after the advent of social media [1]. Scientists and policy makers have recognized its rise and harmful effects as major challenges in the 21st century [2,3]. Misinformation has exacerbated political and religious persecution, hate crimes, climate change, interference in elections, and most recently, the global response to the COVID-19 pandemic [4-8].

Considering people from various social groupings increasingly consume health information via web-based platforms [9], their exposure to health misinformation increases [1,10,11]. Although these platforms can be valuable for health promotion, they can spread false and misleading health information faster than scientific knowledge, raising serious public health concerns [10,12-14]. A recent systematic review found 6 main categories of health misinformation spreading on social media: vaccinations (32%), drugs or smoking (22%), noncommunicable diseases (19%), pandemics (10%), eating disorders (9%), and medical treatments (7%) [10].

Over the past 2 years, the COVID-19 pandemic was associated with what the World Health Organization (WHO) called “an infodemic” [15]. The Director General of the WHO noted [16]:

We’re not just fighting an epidemic; we’re fighting an infodemic, [that] spreads faster and more easily than this virus.

Despite social media organizations’ efforts to limit false health information on the internet [17], COVID-19 misinformation spread unabated [1]. This prompted United Nations agencies to issue warnings against the rapid dissemination of myths, hazardous and untested prevention methods, and fictitious cures that threaten global mitigation plans and put many people’s lives in peril [18-20]. The phenomenon prompted an increase in scientific research about countering and mitigating health misinformation.

Although it dates back to the late 1500s, the term misinformation was selected as the word of the year in 2018 [21]. The Merriam-Webster Dictionary defines it as “false information that is spread, regardless of whether there is intent to mislead.” However, researchers use a growing vocabulary to describe this phenomenon, which includes disinformation, infodemic, malinformation, inaccurate information, misleading information, and conspiracy theories. Inconsistent definitions have also proliferated, which may negatively affect scientific communication and research conceptualization [22]. The objective of this study is to assess definitions of misinformation and related terms used in health research.

## Methods

### Search Strategy

We searched the following 4 electronic databases up till March 6, 2023: MEDLINE (using Ovid), Embase, Cochrane, and Epistemonikos. The search strategy included both controlled vocabularies (eg, MeSH and Emtree) and keywords related to (1) the term “misinformation,” which included “infodemic,” “false news,” “disinformation” and (2) “systematic reviews.” We limited the search to reviews addressing misinformation and published within the past 5 years, starting from January 1, 2017. The final search strategies were developed with the help of an expert librarian after pilot testing with seed articles. The full search strategy for each database is provided in Multimedia Appendix 1.

### Eligibility Criteria

In this study, we included review articles that stated misinformation or related terms as part of their objectives. A review was eligible if it conducted a systematic search of at least 1 database and reported on at least 1 definition for misinformation or related terms. Although the search was not restricted to English reviews, all of the eligible reviews were in English. We included qualitative and quantitative reviews. We excluded all abstracts, nonreviews, narrative reviews without any database search, studies not focusing on misinformation or related terms, studies unrelated to health, and reviews not providing any definition. Textbox 1 summarizes the eligibility criteria used in this scoping review.

**Eligibility criteria for scoping review.**

**Inclusion criteria**

- Review articles
- No restrictions to language
- No restriction to type (qualitative/quantitative)
- Stating misinformation or related terms as part of review objectives
- Systematic search of at least 1 database
- Reported on at least 1 definition for misinformation or related terms
- Related to health

**Exclusion criteria**

- Abstracts and nonreviews
- Studies that do not address misinformation or related terms or with minimal mentioning
- Narrative reviews without any database search
- Reviews not providing any definition
- Unrelated to health

### Study Selection

Three reviewers (IKE, RH, and TH) worked in teams of 2, in duplicate and independently, to screen for potential eligibility of the titles and abstracts of the articles captured by the search. After obtaining the full texts of articles judged as potentially eligible, reviewers included eligible reviews. The principal investigator served as a third independent reviewer for resolving disagreements.

### Data Extraction

Teams of 2 review authors extracted the data from each included review in duplicate and independently. We used a standardized data abstraction form in Excel (Microsoft Corp). We met regularly to discuss progress and resolve any discrepancy through discussion. We abstracted definitions for misinformation and related terms (see Multimedia Appendix 2 [23-61]). We also abstracted the following information for each review: specific health topic, number of searched databases, and the misinformation themes addressed (see Table 1).

**Table 1:** General characteristics of the included systematic reviews (N=41).

Variable		n (%)
**Health topic**		
	Public health emergencies (including COVID-19)	32 (78)
	General health	7 (18)
	Smoking and vaping	1 (2)
	Atopic dermatitis	1 (2)
**Number of databases searched^a^**		
	≥2	32 (78)
	1	9 (22)
**Terms for which a definition was provided^b^**		
	Misinformation	20 (49)
	Disinformation	19 (46)
	Fake news	10 (24)
	Infodemic	24 (59)
	Malinformation	2 (5)
**Misinformation themes addressed^b^**		
	Pathway of misinformation^c^	13 (32)
	Implications of misinformation	8 (20)
	Solutions or interventions	16 (39)
	Epidemiology of misinformation	3 (7)
	Topic-specific examples of misinformation	2 (5)

^a^Google and Google Scholar searches were considered gray literature and not as database.

^b^Numbers add up to more than 41 as some reviews address more than 1 theme and concept.

^c^This theme addresses the mechanisms from the creation to the dissemination of misinformation.

### Data Synthesis

We used content data analysis to identify concepts within the identified definitions. After reading through the definitions several times to obtain a sense of all the definitions, we derived codes that captured key concepts [62]. We used deductive coding to map the definition concepts for each of the misinformation-related terms (see Table 2). Refinements to the language of the definition featured were done after discussions with all authors. When available, the source of the reported definition was noted, and the most used source was tracked (see Table 3).

**Table 2:** Mapping definition concepts to terms of misinformation, disinformation, fake news, infodemic, and malinformation.

Definition concept^a^	Misinformation (20 definitions), n	Disinformation (19 definitions), n	Fake news (10 definitions), n	Infodemic (24 definitions), n	Malinformation (2 definition), n
Information overload	—^b^	—	1	19	—
Valid and invalid info	—	—	—	9	—
False/inaccurate/incorrect	15	13	5	6	—
Fabricated	—	2	4	1	—
Fraudulent	—	—	2	—	—
Accurate	—	—	—	2	2
Clearly unsubstantiated/verifiably false	3	—	3	1	—
Misleading	5	8	2	5	—
Unintentional	7	—	—	—	—
Intentional	5	15	3	—	2
Based on expert opinion	2	—	—	—	—
Used in the wrong context	—	—	—	—	1
Political reasons	—	5	2	—	—
Purpose to instill doubt	—	2	1	—	—
Purpose to manipulate	—	4	2	—	—
Format of official news	—	—	4	—	—
During a health outbreak, epidemic, or crisis	—	—	—	10	—
Epidemic-like spread	—	—	—	4	—
Causes confusion	—	—	—	2	—

^a^The same paper may include more than 1 definition concept.

^b^Not available.

**Table 3:** Source of reported definitions around misinformation and related terms from eligible systematic reviews.

			Misinformation (20 definitions), n/N		Disinformation (19 definitions), n/N		Fake news (10 definitions), n/N		Infodemic (24 definitions), n/N		Malinformation (2 definitions), n/N		Total, n/N
Definition not referenced			5/20		6/19		2/10		1/24		1/2		15/75
Definition referenced+modified			1/20		2/19		1/10		0/24		0/1		4/75
**Definition referenced**			14/20		11/19		7/10		23/24		1/2		56/75
	Merriam-Webster Dictionary	2/14		2/11		0/7		1/23		0/1		5/56	
	WHO^a^	0/14		0/11		0/7		17/23		0/1		17/56	
	Other sources	12/14		9/11		7/7		5/23		1/1		34/56	

^a^WHO: World Health Organization.

## Results

**Figure 1:**
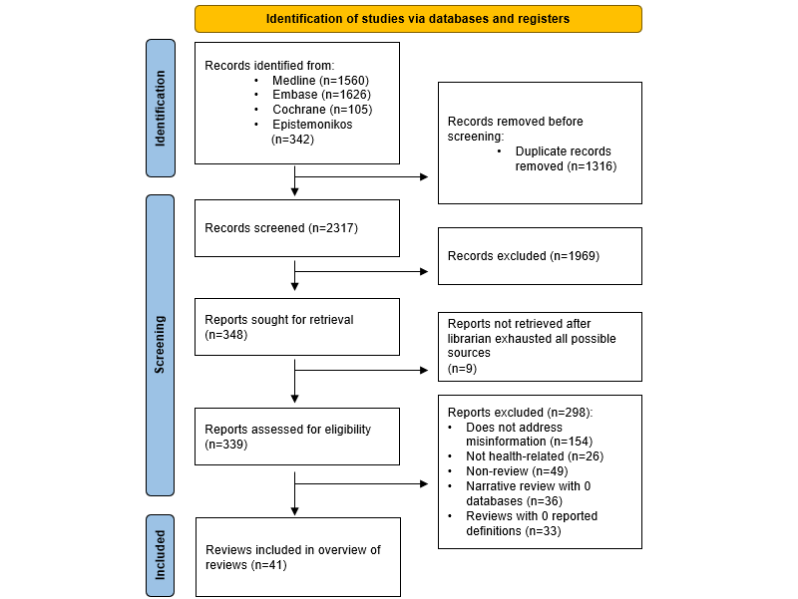
PRISMA (Preferred Reporting Items for Systematic Reviews and Meta-Analyses) 2020 flow diagram.

Out of 3633 articles, the study included 41 systematic reviews [23-61,63]. Figure 1 shows the PRISMA (Preferred Reporting Items for Systematic Reviews and Meta-Analyses) flow diagram detailing the results of the search and selection process (refer to Multimedia Appendix 3 for a PRISMA checklist). Thirty-two (78%) reviews addressed the misinformation-related health of public health emergencies (including COVID-19), with other health topics including general health (18%), smoking and vaping (2%), and atopic dermatitis (2%). Thirty-two (78%) reviews included more than 2 databases for retrieving misinformation-related articles. The reviews addressed many misinformation themes, the most common being solutions/interventions (39%), and the least common being topic-specific examples of misinformation in health topics (5%; Table 1). The definitions extracted from the 41 included reviews consisted of 20 for misinformation, 19 for disinformation, 10 for fake news, 24 for infodemic, and 2 for malinformation (see Multimedia Appendix 2).

The definitions of each misinformation-related term contained definition concepts. Out of the 20 misinformation definitions, 15 definitions mentioned “false/inaccurate/incorrect,” 7 mentioned “unintentional,” and 5 mentioned “intentional.” Out of the 19 definitions for disinformation, 13 mentioned “false/inaccurate/incorrect” and 15 mentioned “intentional.” For fake news, out of the 10 definitions, 5 mentioned “false/inaccurate/incorrect,” 4 mentioned “fabricated,” and 4 mentioned “format of official news.” As for infodemic, out of the 24 definitions, 19 mentioned “information overload,” 9 mentioned “valid and invalid info,” 6 mentioned “false/inaccurate/incorrect” and 10 mentioned “during health outbreak/epidemic/crisis.” Finally, for malinformation, there were 2 definitions and they both mentioned “accurate” and “intentional,” with 1 definition having “used in the wrong context.” For each of the misinformation-related terms, there were additional definition concepts that were less frequently used. This included “fraudulent,” “clearly unsubstantiated/verifiably false,” “misleading,” “based on expert opinion,” “political reasons,” “purpose to instill doubt,” “epidemic-like spread,” and “causes confusion.” Table 2 maps specific concepts of definitions against the different terms being defined.

Out of the 75 definitions used in the reviews, 56 (75%) were referenced from other sources, 15 (20%) were not referenced, and 4 (5%) were referenced from other sources and modified. Out of the 56 referenced definitions, there were 17 (30%) definitions referenced from the WHO and 5 (9%) definitions from the Merriam-Webster Dictionary, with the others being varied resources. The references from WHO all related to the term “infodemic” and accounted for 74% out of the 23 referenced definitions for “infodemic.” None of the reviews provided guidance on operationalizing the reported definitions (eg, further clarifying concepts such as accuracy and intentionality).

## Discussion

### Principal Findings

Misinformation poses a challenge to decision-making. In the field of health, this can affect the clinical decision shared by the caregiver and patient and can affect health care policy making if any of the involved parties is misinformed. In this paper, we assess available definitions of misinformation and related terms used in health research systematic reviews. Across the reviews, there were concepts that were generally agreed upon. Misinformation includes false information that may or may not be intentional. Disinformation, on the other hand, includes intentional dissemination of false information. Fake news includes fabricated, false information disseminated in the format of official news. Infodemic is the information overload that happens in the setting of outbreaks or crises. Finally, malinformation includes accurate information that is used in the wrong context.

The definitions of misinformation and related terms in the literature on health are only 1 part of the extant literature. Fields such as political sciences and media have been well aware of this phenomenon and have studied it extensively. The effect of misinformation on the field of health has become clearer with the recent prominent public health crises including climate change and the COVID-19 pandemic. Our results showed that the definitions used in the field of health are consistent in key concepts but continue to show variability and inconsistency in others.

Definitions of misinformation and related terms vary in definition concepts. One of the clear variabilities in the health literature is related to the intentionality of misinformation. Seven definitions characterized misinformation as unintentional, while 5 definitions characterized it oppositely to be intentional. Another example is the definition of infodemic. Although almost all definitions mentioned the information overload concept, 6 definitions restricted it to false information while 9 definitions attributed it to a mix of valid and invalid information. A third example is the “based on expert opinion” definition concept. Only 2 definitions of misinformation included this concept and this is problematic since expert opinion can be considered a form of evidence. This observed variability and discrepancy highlights the importance of performing a formal consensus process in the field of health to reach a consensus around those definitions.

Definitions of misinformation and related terms contain concepts that require operationalization and clarity. Take for example the concepts of “false/inaccurate/incorrect” and the concept of “intentional.” The label of “false/inaccurate/incorrect” depends on how one defines accuracy and whether the certainty or quality of the evidence is considered. Evidence based on low certainty evidence (eg, a single observational case series) would differ from a higher level of certainty (eg, a systematic review of randomized controlled trials). As for intentionality, it is difficult to assess this as it relates to the intentions of the originator. To reliably assign intentionality to a published piece of false information, a researcher would need to investigate the original source and discern their intentions and purposes. This is not possible for much of the circulating misinformation, particularly those with unverifiable sources. These issues highlight that in addition to the need for a formal consensus process to agree on definitions, these definitions need to be clear and operationalized.

This scoping review has its own strengths and limitations. First, we followed a standard methodology for retrieving relevant articles using 4 distinct databases: Ovid MEDLINE, Embase, Cochrane, and Epistemonikos. Second, we used content data analysis to identify concepts of the retrieved definitions and map them across the misinformation-related terms. This scoping review, however, does not represent all definitions of misinformation in the extant literature, particularly from outside the health field. Also, limiting the search to the past 5 years may have resulted in missing relevant reviews published before that timeframe. However, since reviews published in the last 5 years should have included all the previously published original articles and since we are interested in looking at the current issues and definitions related to misinformation, we are not concerned about this limitation.

As for the implications of this work, our assessment of the definitions highlighted the presence of inconsistencies in the available definitions and some concepts that are difficult to operationalize. Health practitioners need to exert caution before labeling a piece of information as misinformation or any other related terms and only do so after ascertaining few characteristics of the piece of information at hand. A question one should ask is “How certain am I that this information is incorrect?” For disinformation, another question to ask is “How certain am I that this information is disseminated with the knowledge that it is incorrect?” Future work is needed to reach a consensus around clear and operational definitions of misinformation and related terms. This includes performing efforts to reach a formal consensus around those definitions and possibly undergoing qualitative exploratory efforts and interviews with stakeholders.

### Conclusions

Misinformation poses a serious challenge to clinical and policy decision-making in the health field, both of which rely on accurate and reliable information. In this scoping review, we aimed to assess definitions of misinformation and related terms used in the health-related literature. We identified several definitions for misinformation and related terms that showed variability and included concepts that are not conducive to operationalization. Health practitioners need to exert caution before labeling a piece of information as misinformation or any other related term and only do so after ascertaining few characteristics of the piece of information at hand including the accurateness. Additional research is needed to reach a consensus around clear and operational definitions of misinformation and related terms, in order to more effectively study how misinformation affects health policies and research.

## Appendix

Multimedia Appendix 1 Search strategies for MEDLINE (using Ovid), Embase, Cochrane, and Epistemonikos.

Multimedia Appendix 2 Extracted definitions of misinformation and related terms from eligible systematic reviews.

Multimedia Appendix 3 PRISMA (Preferred Reporting Items for Systematic Reviews and Meta-Analyses) checklist.

Multimedia Appendix 4 List of included systematic reviews that address misinformation and related terms in health.

Multimedia Appendix 5 List of excluded systematic reviews that address misinformation and related terms in health but do not provide related definitions.

## Abbreviations

PRISMA: Preferred Reporting Items for Systematic Reviews and Meta-Analyses
WHO: World Health Organization

## Data Availability

All data used for this manuscript can be found in the Multimedia Appendices, including a table of included reviews (Multimedia Appendix 4), a table of excluded reviews (Multimedia Appendix 5), and a table of extracted definitions (Multimedia Appendix 2). RAM affirms that this manuscript is an honest, accurate, and transparent account of the study being reported; that no important aspects of the study have been omitted; and that any discrepancies from the study as planned have been explained.
