# Supplementary material for: Defining Misinformation and Related Terms in Health-Related Literature: Scoping Review
Source: J Med Internet Res. 2023 Aug 9;25:e45731. doi: 10.2196/45731 (PMC10414029; doi:10.2196/45731)
Supplement: Multimedia Appendix 1 [file jmir_v25i1e45731_app1.docx]

**Full Search Strategy 2017 to 2023**

| **Ovid MEDLINE(R) ALL <2017 to March 06, 2023>**  Search date: 06 March 2023 | **Result** |
| --- | --- |
| 1 Disinformation/ or Propaganda/ or Infodemic/ or Deception/ or Superstitions/ | 7494 |
| 2 (misinform* or disinform* or mis-inform* or dis-inform* or malinform* or mal-inform* or infodem* or deceive? or deceit or decept* or propaganda or supersti* or conspiracy theor* or ((information or news) adj1 (disorder or inaccurate or false or mislead* or mis-lead* or fake or low quality or poor quality))).mp. | 24522 |
| 3 1 or 2 | 24522 |
| 4 (exp Meta-Analysis as Topic/ or SYSTEMATIC REVIEW/ or SYSTEMATIC REVIEWS AS TOPIC/ or Meta-Analysis/ or (meta adj analy*).mp. or metaanaly*.mp. or metanaly*.mp. or meta-analysis.pt. or systematic review.pt. or (systematic? adj (review? or overview?)).mp. or exp Review Literature as Topic/ or review.pt. or review.ti. or cochrane.ab. or embase.ab. or (psychlit or psyclit).ab. or (psychinfo or psycinfo).ab. or (cinahl or cinhal).ab. or science citation index.ab. or bids.ab. or cancerlit.ab. or reference list?.ab. or bibliograph?.ab. or hand-search*.ab. or relevant journals.ab. or manual search*.ab. or ((selection criteria or data extraction).ab. and review/)) not (comment/ or letter/ or editorial/ or case report/ or historical article.pt. or review of reported cases.pt. or review,multicase.pt.) | 3476675 |
| 5 3 and 4 | 2795 |
| 6 limit 5 to yr="2017 -Current" | 1560 |

| **Cochrane**  Search date: 06 March 2023 |  |
| --- | --- |
| #1 MeSH descriptor: [Disinformation] this term only | 1 |
| #2 MeSH descriptor: [Propaganda] this term only | 2 |
| #3 MeSH descriptor: [Deception] this term only | 183 |
| #4 MeSH descriptor: [Superstitions] this term only | 11 |
| #5 MeSH descriptor: [Infodemic] this term only | 0 |
| #6 (misinform*):ab,ti,kw OR (disinform*):ab,ti,kw OR (mis-inform*):ab,ti,kw OR (dis-inform*):ab,ti,kw OR (malinform*):ab,ti,kw OR (mal-inform*):ab,ti,kw OR (infodem*):ab,ti,kw OR (deceive?):ab,ti,kw OR (decept*):ab,ti,kw OR (deceit):ab,ti,kw OR (propaganda):ab,ti,kw OR (supersti*):ab,ti,kw OR ("conspiracy theor*"):ab,ti,kw OR (((information OR news) NEAR/1 (disorder OR inaccurate OR false OR mislead* OR mis-lead* OR fake OR "low quality" OR "poor quality")):ti,ab,kw) | 941 |
| #7 MeSH descriptor: [Meta-Analysis as Topic] explode all trees | 1439 |
| #8 MeSH descriptor: [Systematic Review] this term only | 0 |
| #9 MeSH descriptor: [Systematic Reviews as Topic] this term only | 101 |
| #10 MeSH descriptor: [Meta-Analysis] this term only | 0 |
| #11 MeSH descriptor: [Review] this term only | 2 |
| #12 MeSH descriptor: [Review Literature as Topic] 1 tree(s) exploded | 1 |
| #13 ((meta NEXT analy*):ab,ti,kw OR (metaanaly* OR metanaly*):ti,ab,kw OR (meta-analysis):pt OR (“systematic review“):pt OR (systematic? NEXT (review? OR overview?)):ti,ab,kw OR (review):pt OR (review):ti OR (Cochrane):ab or (embase):ab OR (psychlit OR psyclit):ab OR (psychinfo or psycinfo):ab OR (cinahl or cinhal):ab OR ("science citation index"):ab OR (bids):ab OR (cancerlit):ab OR (“reference list?”):ab OR (bibliograph?):ab OR (hand-search*):ab OR ("relevant journals"):ab OR (“manual search*"):ab OR (("selection criteria" OR “data extraction"):ab AND [mh review])) | 86471 |
| #14 (comment):pt OR (letter):pt OR (editorial):pt OR (“case report”):pt OR (“historical article”):pt OR (“review of reported cases”):pt OR (review,multicase):pt | 21289 |
| #15 {OR #1-#6} | 941 |
| #16 {OR #7-#13} | 86471 |
| #17 #16 NOT #14 | 85318 |
| #18 #17 AND #15 with Cochrane Library publication date Between Jan 2017 and Mar 2023 | 105 |

**Embase session results (6 Mar 2023)**

| No. | Query | Results |
| --- | --- | --- |
| #19 | #13 AND #17 AND [2017-2023]/py | 1626 |
| #18 | #13 AND #17 | 3344 |
| #17 | #14 NOT #16 | 3853753 |
| #16 | comment:it OR letter:it OR editorial:it OR 'case report':it OR 'historical article':it OR 'review of reported cases':it OR 'review,multicase':it | 2002989 |
| #15 | #13 AND #14 | 3389 |
| #14 | #3 OR #4 OR #5 OR #6 OR #7 OR #8 OR #9 OR #10 OR #11 OR #12 | 3895164 |
| #13 | #1 OR #2 | 26139 |
| #12 | ('data extraction':ab,ti OR 'selection criteria':ab,ti) AND 'review'/de | 34770 |
| #11 | (manual NEXT/1 search*):ab,ti | 6849 |
| #10 | (reference NEXT/1 list$):ab,ti | 25265 |
| #9 | cochrane:ti,ab OR embase:ti,ab OR psycinfo:ti,ab OR psychinfo:ti,ab OR psychlit:ti,ab OR psyclit:ti,ab OR cinahl:ti,ab OR cinhal:ti,ab OR 'science citation index':ab,ti OR bids:ti,ab OR cancerlit:ti,ab OR bibliograph*:ab,ti OR 'hand-search*':ab,ti OR 'relevant journals':ab,ti | 313371 |
| #8 | 'meta analysis':it OR 'systematic review':it OR review:it OR review:ti | 3466303 |
| #7 | (systematic$ NEXT/1 review$):ti,ab,kw | 337439 |
| #6 | (systematic$ NEXT/1 overview$):ti,ab,kw | 1654 |
| #5 | metaanly*:ti,ab,kw OR metanaly*:ti,ab,kw | 1612 |
| #4 | (meta NEXT/1 analy*):ti,ab,kw | 331363 |
| #3 | 'meta analysis'/exp OR 'systematic review'/de OR 'systematic review (topic)'/de OR 'review'/de OR 'meta analysis (topic)'/de | 3257148 |
| #2 | misinform*:ti,ab,kw OR disinform*:ti,ab,kw OR 'mis inform*':ti,ab,kw OR 'dis inform*':ti,ab,kw OR malinform*:ti,ab,kw OR 'mal inform':ti,ab,kw OR infodem*:ti,ab,kw OR deceive$:ti,ab,kw OR decept*:ti,ab,kw OR deceit:ti,ab,kw OR propaganda:ti,ab,kw OR supersti*:ti,ab,kw OR 'conspiracy theor*':ti,ab,kw OR (((information OR news) NEAR/1 (disorder OR inaccurate OR false OR mislead* OR 'mis lead*' OR fake OR 'low quality' OR 'poor quality')):ti,ab,kw) | 21991 |
| #1 | 'disinformation'/exp OR 'disinformation' OR 'misinformation'/exp OR 'misinformation' OR 'propaganda'/exp OR 'propaganda' OR 'misinformation effect'/exp OR 'misinformation effect' OR 'deception'/exp OR 'deception' OR 'superstition'/exp OR 'superstition' OR 'infodemic'/exp OR 'infodemic' | 17012 |

| **Epistemonikos**  Search date: 06 March 2023 | **Result** |
| --- | --- |
| (((title:("meta analysis" OR "systematic review" OR "systematic overview" OR "review" OR "meta-analysis") OR abstract:("meta analysis" OR "systematic review" OR "systematic overview" OR "review" OR "meta-analysis")) OR (title:(metaanly* OR metanaly*) OR abstract:(metaanly* OR metanaly*)) OR abstract:(cochrane OR embase OR psycinfo OR psychinfo OR psychlit OR psyclit OR cinahl OR cinhal OR "science citation index" OR bids OR cancerlit OR bibliograph* OR "hand-search*" OR "relevant journals" OR "data extraction" OR "selection criteria")) NOT (title:(comment OR letter OR editorial OR "case report" OR "historical article" OR "review of reported cases"))) AND ((title:(misinform* OR disinform* OR "mis inform*" OR "dis inform*" OR malinform*OR "mal inform" OR infodem* OR deceive$ OR decept* OR deceit OR propaganda OR supersti* OR "conspiracy theor*" OR "Information disorder" OR "inaccurate news" OR "inaccurate information" OR "news disorder" OR "false news" OR "false information" OR "mislead* information" OR "mislead* news" OR "mis lead* information" OR "mis lead* news" OR "fake news" OR "fake information" OR "news disorder" OR "low quality news" OR "low quality information" OR "poor quality news" OR "poor quality information") OR abstract:(misinform* OR disinform* OR "mis inform*" OR "dis inform*" OR malinform*OR "mal inform" OR infodem* OR deceive$ OR decept* OR deceit OR propaganda OR supersti* OR "conspiracy theor*" OR "Information disorder" OR "inaccurate news" OR "inaccurate information" OR "news disorder" OR "false news" OR "false information" OR "mislead* information" OR "mislead* news" OR "mis lead* information" OR "mis lead* news" OR "fake news" OR "fake information" OR "news disorder" OR "low quality news" OR "low quality information" OR "poor quality news" OR "poor quality information"))) | 342 |
