# Supplementary material for: Defining Misinformation and Related Terms in Health-Related Literature: Scoping Review
Source: J Med Internet Res. 2023 Aug 9;25:e45731. doi: 10.2196/45731 (PMC10414029; doi:10.2196/45731)
Supplement: Multimedia Appendix 2 [file jmir_v25i1e45731_app2.docx]

## **Extracted definitions of misinformation and related terms from eligible systematic reviews**

| *Misinformation definitions* |
| --- |
| 1. Misinformation, colloquially referred to as “fake news,” is defined as false, inaccurate information that is communicated regardless of an intention to deceive.[23] 2. The information which is fake or misleading and spreads unintentionally is known as misinformation.[24] 3. Misinformation is defined as false information shared by people who have no malicious intention of misleading others. Disinformation is used by governments, militaries, organizations, and individuals to intentionally mislead or manipulate the public.[25] 4. Misinformation is incorrect or misleading information.[26] 5. Misinformation is defined as information that has the features of being false or clearly unsubstantiated, determined based on expert opinion and evidence.[27] 6. Misinformation, in which false information is created and disseminated with the intention of deceiving and/or harming. [28] 7. Misinformation—misleading information created or disseminated without manipulative or malicious intent—can be accessed and shared, especially online.[29] 8. Misinformation involves information that is inadvertently false and is shared without intent to cause harm. [30] 9. Misinformation is incorrect or misleading information.[26] 10. “Misinformation” refers to incorrect information that may or may not be purposefully spread to mislead. [26] 11. Misinformation is defined as information that has the features of being false or clearly unsubstantiated, determined based on expert opinion and evidence.[31] 12. Explicit misinformation (information that is verifiably false based on current scientific evidence) and implicit misinformation (information that misleads the public about the harms and benefits of vaping, e.g., inaccurate and incomplete information), where the primary audience for misinformation is the general public. [31] 13. Misinformation, which is defined as false information, shared without knowledge that it is false. [39] 14. Misinformation, defining it as false information, while subsequent studies expanded its description to include uncertain, vague, and ambiguous information. [32] 15. Misinformation (that is, false or inaccurate information deliberately intended to deceive).[33] 16. The term "misinformation" can refer to both persons who have false beliefs and deceptive information that is purposefully false or inaccurate. Misinformation is deceptive information that is intentionally misleading or incorrect. [34] 17. Misinformation is a piece of false information or inaccurate information that is intentionally created to get more attention from people.[35] 18. Misinformation. Incorrect, misleading or misattributed information circulated without an underlying agenda or intent to harm.[36] 19. Misinformation as any information that counters the current best evidence and expert consensus on the topic.[37] 20. Misinformation is referred to false or inaccurate information deliberately intended to deceive. It originates from rumors, websites and social media, works of fiction, governments, politicians, and vested interests.[38] |
| *Disinformation definitions* |
| 1. Disinformation is defined as the information that is fake or misleading, and unlike misinformation, spreads intentionally. Disinformation campaigns are often seen in a political context where state actors create them for political gains .[24] 2. Disinformation is defined as false information deliberately created and disseminated with malicious intentions.[25] 3. The Merriam-Webster dictionary (2020) defines disinformation as false information deliberately and often covertly spread (as by the planting of rumors) to influence public opinion or obscure the truth. [26] 4. From a more academic view, another definition of disinformation is described as deliberately false or misleading information with its purpose to not always to convince, but to instill doubt. [26] 5. In other words, the term “disinformation” implies intentionality to spread false information to achieve a goal.[26] 6. Disinformation, defined as information that is deliberately false or misleading, often instruments of foreign policy that seek to extend influence and advance geopolitical goals by distorting the information environment and changing how people perceive the world. [27] 7. disinformation, which is the deliberate attempt of individuals, groups and organizations to confuse or manipulate, for economic or political reasons, or simply to disrupt public communication processes.[29] 8. The Merriam-Webster dictionary [2020] defines disinformation as false information deliberately and often covertly spread (as by the planting of rumors) to influence public opinion or obscure the truth. [26] 9. Disinformation is described as deliberately false or misleading information with its purpose to not always to convince, but to instill doubt. [26] 10. In other words, the term, “Disinformation” implies intentionality to spread false information to achieve a goal. [26] 11. Disinformation involves false information knowingly being created and shared to cause harm.[30] 12. Disinformation is fabricated information distributed with the clear intention to mislead, is commonly observed in this era.[39] 13. Unlike misinformation that has no malicious intent, disinformation refers to creating and spreading fake news deliberately to harm an entity, often for political or economic gain.[40] 14. Disinformation: intentional false information or hoax.[41] 15. Disinformation is defined as information that “includes all forms of false, inaccurate, or misleading information designed, presented, and promoted to intentionally cause public harm or for profit.”[32] 16. Disinformation (that is, deliberately misleading or biased information; manipulated narrative or facts; and propaganda).[33] 17. COVID-19 disinformation as scientifically inaccurate claims disseminated with an intent to deceive the public and undermine public health response to the pandemic. In more sophisticated forms of disinformation, the fabricated claims are purposely entangled with semi-accurate information and repackaged as 'alternative facts' to enhance the former's appeal, authenticity, and believability.[42] 18. Disinformation is treated as a part of misinformation.[35] 19. Disinformation. Incorrect, misleading or misattributed information circulated with a specific, often political, agenda. This includes incorrect, misleading or misattributed information, as well as information that is true but artificially amplified, and the manipulation of individuals’ information-seeking, sharing and consumption behaviours.[36] |
| *Fake news definitions* |
| 1. Information overload accompanied by fabricated and fraudulent news. [43] 2. Fake news is the misinformation that is distributed in an official news format. [24] 3. Allcott and Gentzkow defined fake news as “news articles that are intentionally and verifiably false and could mislead readers.” [44] 4. On the other hand, other studies have defined it as “a news article or message published and propagated through media, carrying false information regardless of the means and motives behind it.”. [44] 5. Given this definition, fake news refers to false information that causes an individual to be deceived or doubt the truth, and fake news can only be useful if it actually deceives or confuses consumers. [44] 6. Zhou et al. proposed a broad definition (“Fake news is false news.”) that encompasses false online content and a narrow definition (“Fake news is intentionally and verifiably false news published by a news outlet.” [44] 7. Fake news is fabricated information that mimics news media content, but this does not capture the complexity of the phenomenon, which can include both satire and information created deliberately to mislead as a means to achieve a political or other goal. [30] 8. Such unverified and inaccurate information is generally referred to as fake news, often encompassing misinformation (false information created without any harmful intention), disinformation (false information deliberately created to harm an entity), and malinformation (information based on reality, created to inflict harm on an entity).[40] 9. The authors called fake news the untrue content hidden behind a veneer of legitimacy appropriating the various formats of traditional media: texts, photos, websites, and videos. This manipulation can generate several combinations - in public health, for example, the effect is harmful since society aims to seek information which generates health and well-being. The Lexico dictionary (2022), by Oxford University Press, defines fake news as false information transmitted or published as news for fraudulent or politically motivated purposes. [45] 10. Fake news is a modified version of original news which is used to misguide the people or manipulate public opinion using traditional mass media and online social media). It is also known as fabricated information which differs in organizational procedure or purpose but looks similar to news media content). It can be misleading or dangerous when it is out of context and original sources. It is used to describe phony press releases, hoaxes, and spam since there is no official definition. These kinds of news are unreliable and create misconceptions among the people. [35] |
| *Infodemic definitions* |
| 1. Infodemic is short for “information x”, depicting the rapid spread and amplification of vast amounts of valid and invalid information. [46] 2. An infodemic is defined by the WHO as ‘too much information including false or misleading information in digital and physical environments during a disease outbreak. [47] 3. As coined by the WHO, the pandemic has also spawned an “infodemic”, the plethora of information created during a health crisis, which is misleading and untrue [48] 4. The WHO, whose primary role is to direct international health within the United Nations’ system and to lead partners in global health responses defines the infodemic as follows (literal quote):“*An infodemic is too much information including false or misleading information in digital and physical environments during a disease outbreak. It causes confusion and risk-taking behaviours that can harm health. It also leads to mistrust in health authorities and undermines the public health response. An infodemic can intensify or lengthen outbreaks when people are unsure about what they need to do to protect their health and the health of people around them. With growing digitization—an expansion of social media and internet use—information can spread more rapidly. This can help to more quickly fill information voids but can also amplify harmful messages. Infodemic management is the systematic use of risk- and evidence-based analysis and approaches to manage the infodemic and reduce its impact on health behaviours during health emergencies. Infodemic management aims to enable good health practices through 4 types of activities: Listening to community concerns and questions, Promoting understanding of risk and health expert advice, Building resilience to misinformation, Engaging and empowering communities to take positive action.”* [49] 5. An “infodemic” is an overabundance of information—in some cases accurate and in others not—that is disseminated during an epidemic and that spreads among humans in an epidemic-like manner through digital and physical information systems. [25] 6. The World Health Organization has declared the ‘infodemic’ – an overabundance of information and rapid spread of misleading or fabricated news, images and videos – as one of the greatest threats to global health. [50] 7. The term infodemic was coined in 2003 by David Rothkopf, a writer for Washington Post.It has different definitions, varying from "a few facts, mixed with fear, speculation, and rumor, amplified and relayed swiftly worldwide by modern information technologies" to "a rapid and far-reaching spread of both accurate and inaccurate information about something, such as a disease" by Merriam-Webster dictionary. [51] 8. The World Health Organisation (WHO) had warned the public of an ‘infodemic’, described as ‘an overabundance of information, some accurate and some not, that makes it hard for people to find trustworthy sources and reliable guidance when they need it. [52] 9. The pandemic caused the emergence of a phenomenon, defined as an overabundance of information, called infodemic which may or may not be true, that spread similarly to an epidemic through digital and physical information systems, making it difficult to obtain reliable sources and guidance when necessary. [28] 10. The concept of infodemic adopted was that of the WHO, which describes it as a superabundance of information – some accurate and some not – which occurs during an epidemic and can spread through physical and digital means, making it difficult to access reliable sources and reliable guidance, when necessary.[28] 11. An infodemic is defined as the spread of too much information—including false or misleading information in the digital and physical environments—during a disease outbreak. [29] 12. “infodemic”, which has been recently defined as “an overabundance of information – some accurate and some not – that makes it hard for people to find trustworthy sources and reliable guidance when they need it.” [50] 13. The term “infodemic” was coined by Gunther Eysenbach. It was adopted by the WHO in a report on 2 February 2020 to describe “an over-abundance of information –some accurate and some not –that makes it hard for people to find trustworthy sources and reliable guidance when they need it”, when massive misinformation and conspiracy theories relating to COVID-19 were circulating widely on the internet. [53] 14. “Infodemic”: “an over-abundance of information—some accurate and some not—that makes it hard for people to find trustworthy sources and reliable guidance when they need it.” [54] 15. People’s human information behavior (HIB) during the crisis was seriously challenged by the dissemination of false information in a context that has often been defined as infodemics.[55] 16. According to the World Health Organization, infodemic is exposure to too much online and offline information, which can also occur during an epidemic or pandemic disease outbreak. This phenomenon causes confusion and high-risk behaviors during pandemics and can negatively affect public health. [56] 17. An excessive amount of information shared physically and digitally about the virus, disease, treatment, Standard Operating Procedures (SOP), lockdowns and vaccines, among others – a phenomenon known as infodemic. This information is typically unreliable, unverified, spreads rapidly, and thus making it difficult for the public to make informed decisions and solutions difficult to achieve. [40] 18. Infodemics tend to spread widely within a short time, the World Health Organization (WHO) attempted to define the term as the outbreak of context-based reliable and unreliable information during epidemics.[57] 19. The WHO defined it as an overabundance of information, true and false, that makes it difficult to find reliable sources and recommendations.[41] 20. Infodemic, which is defined as an overabundance of information, only some of which is accurate, that makes it difficult for people to find trustworthy sources and reliable guidance.[32] 21. Infodemic, involves a torrent of online information containing either false and misleading information or accurate content.[33] 22. Infodemics is defined as “an overburden of information – some accurate and some not – that makes it hard for people to find trustworthy source and reliable guidance when they need it.” [58] 23. An infodemic is “too much information including false or misleading information in digital and physical environments during a disease outbreak.” [59] 24. WHO defines an infodemic as “too much information, including false or misleading information, in digital and physical environments during a disease outbreak”. The term infodemic, a word blend of information and epidemic, was first used during the 2003 severe acute respiratory syndrome (SARS) outbreak to describe the ways in which new information technologies complicated risk communication during that event. [36] |
| *Malinformation definition* |
| 1. Malinformation, is accurate information that is used in different contexts to spread hatred or abuse of a person or a particular group .[24] 2. Malinformation: true information used to harm.[41] |
| *Relationship between infodemic and misinformation* |
| 1. The term infodemic has been used to outline the hazards of misinformation during the management of disease outbreaks, since it could negatively affect the social response to the pandemic.[39] |
| *Key difference between misinformation and disinformation* |
| 1. The key difference between misinformation and disinformation is that misinformation is accidental falsehood and disinformation is deliberate falsehood.[27] 2. A distinction has been made between misinformation, defined as incorrect or false information that is shared without the intent to harm, and disinformation, defined as incorrect or false information that is shared with the aim of causing harm.[60] 3. Content that is inadvertently misleading with no intention of hurting others (misinformation) and another that is spread with an intent to cause harm (disinformation).[42] 4. The differentiation between disinformation and misinformation is further complicated by the difficulty of determining the intent of those who peddle falsehood about COVID-19. [42] 5. Despite their similarities, they differ slightly in terms of usage contexts, degrees of incorrectness as well as the functions of serving in various propagation scenarios. [35] 6. Misinformation is inaccurate information that is unintentionally presented as fact, while disinformation involves deliberately spreading false information to cause harm. [61] |
