# Supplementary material for: Defining Misinformation and Related Terms in Health-Related Literature: Scoping Review
Source: J Med Internet Res. 2023 Aug 9;25:e45731. doi: 10.2196/45731 (PMC10414029; doi:10.2196/45731)
Supplement: Multimedia Appendix 4 [file jmir_v25i1e45731_app4.docx]

## **List of included systematic reviews that address misinformation and related terms in health**

| 1. Joseph, A.M., et al., COVID-19 Misinformation on Social Media: A Scoping Review. Cureus, 2022. 14(4): p. e24601.  2. Muhammed, T.S. and S.K. Mathew, The disaster of misinformation: a review of research in social media. International Journal Of Data Science And Analytics, 2022. 13(4): p. 271-285.  3. Clemente-Suarez, V.J., et al., Mis-Dis Information in COVID-19 Health Crisis: A Narrative Review. International Journal of Environmental Research & Public Health [Electronic Resource], 2022. 19(9): p. 27.  4. Patel, S.S., et al., The Landscape of Disinformation on Health Crisis Communication During the COVID-19 Pandemic in Ukraine: Hybrid Warfare Tactics, Fake Media News and Review of Evidence. Jcom Journal of Science Communication, 2020. 19(5).  5. Janmohamed, K., et al., Interventions to Mitigate COVID-19 Misinformation: A Systematic Review and Meta-Analysis. Journal of Health Communication, 2021. 26(12): p. 846-857.  6. Delgado, C.E., et al., COVID-19 infodemic and adult and elderly mental health: a scoping review. Revista Da Escola de Enfermagem Da Usp, 2021. 55: p. e20210170.  7. Magarini, F.M., et al., Irrational Beliefs about COVID-19: A Scoping Review. International Journal of Environmental Research & Public Health [Electronic Resource], 2021. 18(19): p. 22.  8. Wang, Y., et al., Systematic Literature Review on the Spread of Health-related Misinformation on Social Media. Social Science & Medicine, 2019. 240: p. 112552.  9. Sonny, S.P., et al., The landscape of disinformation on health crisis communication during the COVID-19 pandemic in Ukraine: hybrid warfare tactics, fake media news and review of evidence. JCOM, Journal of science communication, 2020. 19(5).  10. Janmohamed, K., et al., Interventions to Mitigate Vaping Misinformation: A Meta-Analysis. Journal of Health Communication, 2022. 27(2): p. 84-92.  11. Czerniak, K., et al., A scoping review of digital health interventions for combating COVID-19 misinformation and disinformation. Journal of the American Medical Informatics Association, 2023. 30(4): p. 752-760.  12. Borges do Nascimento, I.J., et al., Infodemics and health misinformation: a systematic review of reviews. Bulletin of the World Health Organization, 2022. 100(9): p. 544-561.  13. Ravichandran, B.D. and P. Keikhosrokiani, Classification of Covid-19 misinformation on social media based on neuro-fuzzy and neural network: A systematic review. Neural Computing and Applications, 2023. 35(1): p. 699-717.  14. Sanaullah, A., et al., Applications of machine learning for COVID-19 misinformation: a systematic review. Social Network Analysis and Mining, 2022. 12(1): p. 94.  15. Tomes, N. and M. Parry, What are the historical roots of the COVID-19 infodemic? Lessons from the past. 2022.  16. Vraga, E.K., et al., HPV and HBV vaccine hesitancy, intention and uptake in the era of social media and COVID-19: A review. medRxiv, 2023: p. 2023.01. 25.23285015.  17. Zhao, S., et al., The Prevalence, Features, Influencing Factors, and Solutions for COVID-19 Vaccine Misinformation: Systematic Review. JMIR Public Health and Surveillance, 2023. 9(1): p. e40201.  18. Tentolouris, A., et al., COVID-19: time to flatten the infodemic curve. Clinical & Experimental Medicine, 2021. 21(2): p. 161-165.  19. Balakrishnan, V., et al., Infodemic and fake news–A comprehensive overview of its global magnitude during the COVID-19 pandemic in 2021: A scoping review. International Journal of Disaster Risk Reduction, 2022: p. 103144.  20. Casino, G., Communication in times of pandemic: information, disinformation, and provisional lessons from the coronavirus crisis. Gaceta Sanitaria, 2022. 36: p. S97-S104.  21. Kemei, J., et al., A scoping review of COVID-19 online mis/disinformation in Black communities. Journal of Global Health, 2022. 12.  22. Rocha, Y.M., et al., The impact of fake news on social media and its influence on health during the COVID-19 pandemic: a systematic review. Journal of Public Health, 2021: p. 1-10.  23. Kim, B., et al., A systematic review on fake news research through the lens of news creation and consumption: Research efforts, challenges, and future directions. PLoS ONE [Electronic Resource], 2021. 16(12): p. e0260080.  24. Raquel, C.P., et al., Scientific ways to confront covid-19 fake news. Saúde e Sociedade, 2022. 31.  25. Chowdhury, N., A. Khalid, and T.C. Turin, Understanding misinformation infodemic during public health emergencies due to large-scale disease outbreaks: a rapid review. Journal of Public Health, 2021: p. 1-21.  26. Choukou, M.A., et al., COVID-19 infodemic and digital health literacy in vulnerable populations: A scoping review. Digital Health, 2022. 8: p. 20552076221076927.  27. Ferreira Caceres, M.M., et al., The impact of misinformation on the COVID-19 pandemic. Aims Public Health, 2022. 9(2): p. 262-277.  28. Corinti, F., D. Pontillo, and D. Giansanti, COVID-19 and the Infodemic: An Overview of the Role and Impact of Social Media, the Evolution of Medical Knowledge, and Emerging Problems. Healthcare, 2022. 10(4): p. 14.  29. O'Connor, C. and M. Murphy, Scratching the surface: a review of online misinformation and conspiracy theories in atopic dermatitis. Clinical & Experimental Dermatology, 2021. 46(8): p. 1545-1547.  30. Pian, W., J. Chi, and F. Ma, The causes, impacts and countermeasures of COVID-19 "Infodemic": A systematic review using narrative synthesis. Information Processing & Management, 2021. 58(6): p. 102713.  31. Sasidharan, S., et al., COVID-19: Pan(info)demic. Turk Anestezi Ve Reanimasyon Dergisi, 2020. 48(6): p. 438-442.  32. La Bella, E., C. Allen, and F. Lirussi, Communication vs evidence: What hinders the outreach of science during an infodemic? A narrative review. Integrative Medicine Research, 2021. 10(4): p. 100731.  33. Oxman, M., et al., Quality of information in news media reports about the effects of health interventions: Systematic review and meta-analyses. F1000Research, 2021. 10: p. 433.  34. Montesi, M., Human information behavior during the Covid-19 health crisis. A literature review. Library & Information Science Research, 2021. 43(4): p. 101122.  35. Aslani, N., et al., Infodemic Challenges During COVID-19 Pandemic and the Strategies to Deal with Them: A Review Article. Archives of Clinical Infectious Diseases, 2022. 17(1).  36. Bam, N.E., Strategies to address conspiracy beliefs and misinformation on COVID-19 in South Africa: A narrative literature review. Health SA Gesondheid (Online), 2022. 27: p. 1-8.  37. Sharma, L.D., et al., Infodemics during era of COVID-19 pandemic: A review of literature. Journal of family medicine and primary care, 2022. 11(8): p. 4236-4239.  38. Skafle, I., et al., Misinformation about COVID-19 vaccines on social media: rapid review. Journal of medical Internet research, 2022. 24(8): p. e37367.  39. Gabarron, E., S.O. Oyeyemi, and R. Wynn, COVID-19-related misinformation on social media: a systematic review. Bulletin of the World Health Organization, 2021. 99(6): p. 455-463A.  40. Whitehead, H.S., et al., A systematic review of communication interventions for countering vaccine misinformation. Vaccine, 2023.  41. Eysenbach, G., Infodemiology and infoveillance: framework for an emerging set of public health informatics methods to analyze search, communication and publication behavior on the Internet. Journal of medical Internet research, 2009. 11(1): p. e1157. |
| --- |
