# Supplementary material for: Defining Misinformation and Related Terms in Health-Related Literature: Scoping Review
Source: J Med Internet Res. 2023 Aug 9;25:e45731. doi: 10.2196/45731 (PMC10414029; doi:10.2196/45731)
Supplement: Multimedia Appendix 5 [file jmir_v25i1e45731_app5.docx]

## **List of excluded systematic reviews that address misinformation and related terms in health but do not provide related definitions**

| 1. O'Connor C, O'Grady C, Murphy M. Spotting fake news: a qualitative review of misinformation and conspiracy theories in acne vulgaris. Clinical & Experimental Dermatology. 2022;18:18. 2. O'Connor C, Rafferty S, Murphy M. A qualitative review of misinformation and conspiracy theories in skin cancer. Clinical & Experimental Dermatology. 2022;05:05. 3. Bierwiaczonek K, Gundersen AB, Kunst JR. The role of conspiracy beliefs for COVID-19 health responses: A meta-analysis. Current Opinion in Psychology. 2022;46:101346. 4. Ripp T, Roer JP. Systematic review on the association of COVID-19-related conspiracy belief with infection-preventive behavior and vaccination willingness. BMC psychology. 2022;10(1):66. 5. Roche D, Murphy M, O'Connor C. A qualitative analysis of online misinformation and conspiracy theories in psoriasis. Clinical & Experimental Dermatology. 2022;47(5):949-52. 6. Lazic A, Zezelj I. A systematic review of narrative interventions: Lessons for countering anti-vaccination conspiracy theories and misinformation. Public Understanding of Science. 2021;30(6):644-70. 7. Yeung AWK, Tosevska A, Klager E, Eibensteiner F, Tsagkaris C, Parvanov ED, et al. Medical and Health-Related Misinformation on Social Media: Bibliometric Study of the Scientific Literature. Journal of Medical Internet Research. 2022;24(1):e28152. 8. Suarez-Lledo V, Alvarez-Galvez J. Prevalence of Health Misinformation on Social Media: Systematic Review. Journal of Medical Internet Research. 2021;23(1):e17187. 9. Walter N, Brooks JJ, Saucier CJ, Suresh S. Evaluating the Impact of Attempts to Correct Health Misinformation on Social Media: A Meta-Analysis. Health Communication. 2021;36(13):1776-84. 10. Patev AJ, Hood KB. Towards a better understanding of abortion misinformation in the USA: a review of the literature. Culture, Health & Sexuality. 2021;23(3):285-300. 11. Pradeep T, Ravipati A, Melachuri S, Fu R. More than just a stye: identifying seasonal patterns using google trends, and a review of infodemiological literature in ophthalmology. Orbit. 2022:1-8. 12. Mirza SA, Sheikh AAE, Barbera M, Ijaz Z, Javaid MA, Shekhar R, et al. COVID-19 and the Endocrine System: A Review of the Current Information and Misinformation. Infectious Disease Reports. 2022;14(2):184-97. 13. Salehinejad S, Jangipour Afshar P, Borhaninejad V. Rumor surveillance methods in outbreaks: A systematic literature review. Health Promotion Perspectives. 2021;11(1):12-9. 14. Geronikolou S, Chrousos G. COVID-19-Induced Fear in Infoveillance Studies: Pilot Meta-analysis Study of Preliminary Results. JMIR Formative Research. 2021;5(2):e21156. 15. Upadhyay MK, Maroof KA. Understanding the emerging and reemerging terminologies amid the COVID-19 pandemic. Journal of Family Medicine & Primary Care. 2020;9(12):5881-7. 16. Gruber A, Ghiringhelli M, Edri O, Abboud Y, Shiti A, Shaheen N, et al. Literature Review and Knowledge Distribution During an Outbreak: A Methodology for Managing Infodemics. Academic Medicine. 2021;96(7):1005-9. 17. Majid U, Wasim A, Bakshi S, Truong J. Knowledge, (mis-)conceptions, risk perception, and behavior change during pandemics: A scoping review of 149 studies. Public Understanding of Science. 2020;29(8):777-99. 18. Favaloro EJ, Thachil J. Reporting of D-dimer data in COVID-19: some confusion and potential for misinformation. Clinical Chemistry & Laboratory Medicine. 2020;58(8):1191-9. 19. Oelke M, Bschleipfer T, Höfner K. Fake News BPH – what is really true! Urologe. 2019;58(3):271-83. 20. Akem Dimala C, Kadia BM, Nguyen H, Donato A. Community and provider acceptability of the COVID-19 vaccine: A systematic review and meta-analysis. Tropical Medicine and International Health. 2021;26(SUPPL 1):246-7. 21. Ahiagba P, Alexis O, Worsley AJ. Factors influencing black men and their partners' knowledge of prostate cancer screening: a literature review. British Journal of Nursing. 2017;26(18):S14-S21. 22. Delgado-Lopez PD, Corrales-Garcia EM. Influence of Internet and Social Media in the Promotion of Alternative Oncology, Cancer Quackery, and the Predatory Publishing Phenomenon. Cureus. 2018;10(5):e2617. 23. Kamal A, Hodson A, Pearce JM. A rapid systematic review of factors influencing covid‐19 vaccination uptake in minority ethnic groups in the uk. Vaccines. 2021;9(10). 24. Lee CP, Holmes T, Neri E, Kushida CA. Deception in clinical trials and its impact on recruitment and adherence of study participants. Contemporary Clinical Trials. 2018;72:146-57. 25. Rocha YM, de Moura GA, Desiderio GA, de Oliveira CH, Lourenco FD, de Figueiredo Nicolete LD. The impact of fake news on social media and its influence on health during the COVID-19 pandemic: a systematic review. Journal of Public Health. 2021:1-10. 26. Seaman AT, Stone AM. Little White Lies: Interrogating the (Un)acceptability of Deception in the Context of Dementia. Qualitative Health Research. 2017;27(1):60-73. 27. Staccini P, Lau AYS, Section Editors for the IYSoCHI. Consumer Informatics and COVID-19 Pandemics: Challenges and Opportunities for Research. Yearbook of medical informatics. 2021;30(1):210-8. 28. Adu P, Poopola T, Medvedev ON, Collings S, Mbinta J, Aspin C, et al. Implications for COVID-19 vaccine uptake: A systematic review. Journal of Infection and Public Health. 2023. 29. Finnegan P, Murphy M, O’Connor C. # corticophobia: a review on online misinformation related to topical steroids. Clinical and Experimental Dermatology. 2023 Feb;48(2):112-5. 30. Nan X, Wang Y, Thier K. Why people believe health misinformation and who are at risk? A systematic review of individual differences in susceptibility to health misinformation. Social Science & Medicine. 2022 Oct 21:115398. 31. Sharma PR, Wade KA, Jobson L. A systematic review of the relationship between emotion and susceptibility to misinformation. Memory. 2023 Jan 2;31(1):1-21. 32. Tsamakis K, Tsiptsios D, Stubbs B, Ma R, Romano E, Mueller C, Ahmad A, Triantafyllis AS, Tsitsas G, Dragioti E. Summarising data and factors associated with COVID-19 related conspiracy theories in the first year of the pandemic: a systematic review and narrative synthesis. BMC psychology. 2022 Nov 1;10(1):244. 33. Yong-Hing CJ, Gordon PB, Appavoo S, Fitzgerald SR, Seely JM. Addressing misinformation about the Canadian breast screening guidelines. Canadian Association of Radiologists Journal. 2023 May;74(2):388-97. |
| --- |
